# Expression and Function of the Protein Tyrosine Phosphatase Receptor J (*PTPRJ*) in Normal Mammary Epithelial Cells and Breast Tumors

**DOI:** 10.1371/journal.pone.0040742

**Published:** 2012-07-17

**Authors:** Chanel E. Smart, Marjan E. Askarian Amiri, Ania Wronski, Marcel E. Dinger, Joanna Crawford, Dmitry A. Ovchinnikov, Ana Cristina Vargas, Lynne Reid, Peter T. Simpson, Sarah Song, Christiane Wiesner, Juliet D. French, Richa K. Dave, Leonard da Silva, Amy Purdon, Megan Andrew, John S. Mattick, Sunil R. Lakhani, Melissa A. Brown, Stuart Kellie

**Affiliations:** 1 School of Chemistry and Molecular Biosciences, The University of Queensland, Brisbane, Queensland, Australia; 2 Centre for Clinical Research, The University of Queensland, Brisbane, Queensland, Australia; 3 Queensland Institute of Medical Research, Brisbane, Queensland, Australia; 4 Institute for Molecular Biosciences, The University of Queensland, Brisbane, Queensland, Australia; 5 School of Medicine, The University of Queensland, Brisbane, Queensland, Australia; 6 University of Queensland, Department of Anatomical Pathology, Brisbane, Queensland, Australia; Baylor College of Medicine, United States of America

## Abstract

The protein tyrosine phosphatase receptor J, PTPRJ, is a tumor suppressor gene that has been implicated in a range of cancers, including breast cancer, yet little is known about its role in normal breast physiology or in mammary gland tumorigenesis. In this paper we show that *PTPRJ* mRNA is expressed in normal breast tissue and reduced in corresponding tumors. Meta-analysis revealed that the gene encoding PTPRJ is frequently lost in breast tumors and that low expression of the transcript associated with poorer overall survival at 20 years. Immunohistochemistry of PTPRJ protein in normal human breast tissue revealed a distinctive apical localisation in the luminal cells of alveoli and ducts. Qualitative analysis of a cohort of invasive ductal carcinomas revealed retention of normal apical PTPRJ localization where tubule formation was maintained but that tumors mostly exhibited diffuse cytoplasmic staining, indicating that dysregulation of localisation associated with loss of tissue architecture in tumorigenesis. The murine ortholog, *Ptprj,* exhibited a similar localisation in normal mammary gland, and was differentially regulated throughout lactational development, and in an *in vitro* model of mammary epithelial differentiation. Furthermore, ectopic expression of human PTPRJ in HC11 murine mammary epithelial cells inhibited dome formation. These data indicate that PTPRJ may regulate differentiation of normal mammary epithelia and that dysregulation of protein localisation may be associated with tumorigenesis.

## Introduction

Loss of hetreozygosity (LOH) studies have implicated the protein tyrosine phosphatase receptor J (*PTPRJ, DEP-1, PTP-η, CD148*) gene in the development of human meningioma [1], colon, lung and breast cancers, and quantitative trait analysis in mouse identified the mouse *Ptprj* orthologue as the sole candidate gene for the murine colon cancer susceptibility locus (*Scc1*) [2]. A reduction in the proliferation, survival and tumorigenicity of several cell types upon ectopic expression of PTPRJ further suggests a tumor suppressor role for this protein [3], [4], [5], [6], [7], [8]. Typical tumor suppressor gene ‘loss of function’ can result from loss or alteration of protein function, epigenetic silencing, RNA interference or post-translational modifications, or dysregulation by noncoding (nc) RNA [9], [10].

*PTPRJ* encodes a receptor-like protein tyrosine phosphatase that can attenuate intracellular signals mediated by MAPK, p21Ras and Akt kinases [3], [5], [6], [11], [12]. The targets of PTPRJ include p120 catenin, Gab1, Met [13], [14], PDGF β-receptor [15], VEGFR2 [12] EGFR [16] and p85α [17]. More recently PTPRJ was identified in phosphotome screening as a potent negative regulator of Akt activation in Ras-mutated cancer cells [18] and it directly desphosphorylates ERK1/2 [19]. However, PTPRJ also activates *src* family members by dephosphorylating the negative regulatory carboxyterminal phosphotyrosine, indicating a positive role in some *src* signalling pathways [20].

Since breast cancer is the most common female cancer and the leading cause of cancer-related death among women, identifying genes involved in this process is of significant interest. The role of *PTPRJ* in breast cancer and in normal breast biology is not well understood. Genome wide association (GWA) studies has identified a specific *PTPRJ* breast cancer protective haplotype, however the causal SNP has not yet been determined [21]. *PTPRJ* LOH has, however, been reported in a small number of breast tumors [2] and allele-specific *PTPRJ* LOH suggests the existence of a putative cancer resistance *PTPRJ* SNP (A1176C) that is more frequently lost in tumors with *PTPRJ* LOH. This non-conservative substitution in the second fibronectin (FN III) repeat is hypothesized to lead to a conformational change, potentially altering protein function.

These studies highlight the possibility that, even with normal protein expression levels, a PTPRJ SNP could affect protein conformation, leading to altered PTPRJ function. A SNP linked to thyroid cancer [22] led to changes in the 8^th^ FN III repeat resulting in the loss of plasma membrane localisation and loss of growth inhibitory activity of PTPRJ [23]. In addition, the interaction between PTPRJ and the tight junction proteins occludin and ZO-1 in MCF10A breast epithelial cells and the effect of overexpression on transepithelial resistance in MDCK cells indicates an important role in the regulation of epithelial barrier function [24]. The localisation of PTPRJ is clearly an important feature of its function, yet to date, this has not been investigated in the normal breast or breast cancer samples. A further possibility is that a disease-associated SNP or somatic mutation in the *PTPRJ* locus may affect the function of a noncoding RNA that originates from the same locus. Indeed, the majority of SNPs occur within noncoding regions of the genome and many noncoding RNAs are involved in disease etiology [25].

Several molecules implicated in breast tumorigenesis, including p53, BRCA1 and ATM, play an important role in normal mammary gland development [26], [27], [28], [29], [30]. Understanding the role of such molecules in normal development is critical to understanding how changes in their expression or function contribute to tumorigenesis. The mechanisms underlying the regulation of *PTPRJ* expression have not yet been explored, nor whether its dysregulation may contribute clinically to breast cancer progression, as would be expected from a tumor suppressor gene. In this study, we therefore investigated its genomic alteration, transcript and protein expression and localization in large cohorts of breast cancer samples, and in a mouse model of mammary gland development.

## Materials and Methods

### Cancer Survey Panel

Comprehensive pathology reports and details on the tissues comprising the TissueScan Cancer Survey Panel 384-I (containing two identical sets of 381 tissues covering twenty two different cancers) can be found on the supplier’s homepage (http://www.origene.com/qPCR/Tissue-qPCR-Arrays.aspx).

### Metaanalyses

The NKI-295 cohort gene expression data set [31] was downloaded from the Netherlands Cancer Institute website (http://bioinformatics.nki.nl/data.php). The Borg-395 cohort data set containing gene expression and tumor DNA copy number data (BAC array CGH) [32] was downloaded from the NCBI Gene Expression Omnibus (http://www.ncbi.nlm.nih.gov/geo/) via the series accession number GSE22133. The normalized gene expression profiles were used for both NKI-295 and Borg-395 data sets as described in original publications.

The molecular subtypes (Basal, HER2, Luminal A, Luminal B and Normal-like) of breast cancer were classified using the single sample predictor method developed by Hu *et al*. [33] in the two data sets. Each sample was assigned into a subtype based on the highest spearman rank correlation to the 306 gene centroids. Cases that did not have any correlation greater than 0.1 to each of the centroids were recorded as “nonClassified” as described by Sorlie *et al* [34]. Either analysis of variance (ANOVA) or the Kruskal-Wallis test was employed to determine differences in *PTPRJ* expression between the molecular subtypes or histological grade of the tumor. A 5% significant level was used. A Tukey post hoc test [35] was used to detect the difference between each pair of subtypes if the result of the ANOVA test was statistically significant. In the survival analysis, high/low *PTPRJ*-expressing groups were defined if gene expression of *PTPRJ* was higher than the 75-percent quantile or lower than the 25-percent quantile. Kaplan-Meier survival curves between the high/low groups were visualized and the difference between the two curves was tested using a log-rank test [36]. The analysis was performed in R version 2.10.1 (http://www.r-project.org/).

For genomic DNA the segmental DNA copy number estimation analysis was performed using the circular binary segmentation (CBS) method [37], [38], [39] implemented using the Bioconductor package DNAcopy [38]. The significance level of 0.01 was used for the test to accept change-points and only segmentations containing more than 4 BAC probes were used in the further analysis. The segmentation mean above 0.1 was defined as gain in DNA copy number, below −0.1 as losses, and in between as copy neutral, as defined by the authors [32]. Data was analysed and a frequency plot of gains and losses across the whole genome recapitulated those from the original study (data not shown). The copy number status of the *PTPRJ* gene locus at position 47,958,689–48,146,246 on chromosome 11 was determined in all breast cancers analysed and was also stratified according to molecular subtype, defined by gene expression profiling data from the same tumors. The goodness of fit tests for multinomial distribution [40] were used to detect whether the observed pattern of genomic changes for each subtype is similar to the one for the overall samples given a significant level of 0.05.

### Human Tissue

Fresh frozen sections were made from normal human breast tissue taken from 2 individual mastectomy samples and from 27 breast tumor samples from consenting patients undergoing surgery at the Royal Brisbane and Women’s Hospital. Snap frozen normal tissue pieces were also used for RNA extraction. Diagnostic information about these cases was retrieved from hospital pathology records. This work was approved by human ethics committee of the University of Queensland and the Royal Brisbane and Women’s Hospital.

### Mouse Tissue

Abdominal mammary glands from CBA x C57Bl6 mice were extracted and processed as previously described [41]. Tissues were taken from nulliparous (virgin) animals (n = 4), day 14 of pregnancy (n = 4), day 1 of lactation (n = 3) and day 2 of involution (n = 4). *In situ* hybridisation (see below) was performed on mammary glands extracted from pregnant BALBc mice. All animal work was conducted according to relevant national guidelines approved by The University of Queensland Animal Ethics Committee, approval number: BIOC/393/05/URG.

### Cell Culture

MDAMB-231, MDA-MB-468, T-47D, MCF7, ZR751 and SVCT cells were obtained through and cultured according to the supplier’s recommendations as previously described [42]. MCF10A cells were cultured in RPMI (Gibco, Invitrogen) containing 5% horse serum (Gibco, Invitrogen), 10 µg/mL insulin (Sigma-Aldrich), 20 ng/mL Epidermal Growth Factor (EGF; Becton Dickinson) and antibiotic/antimycotic (Gibco, Invitrogen). HC11 cells (from Dr. Chris Ormandy, Garvan Institute, Sydney, Australia) were maintained in RPMI 1640 medium (Gibco, Invitrogen) with 10% Fetal Calf Serum (FCS) (Gibco, Invitrogen), 5 µg/mL insulin, and 10 ng/mL EGF.

### Immunohistochemistry (IHC) and Immunofluorescence

Human breast sections were fixed in acetone and processed using the DakoCytomation Envision®+ Dual Link System-HRP (DAB+) according to manufacturers’ instructions. IHC slides were scanned on an Aperio Scanscope XT and images were taken using supplied software. For immunofluorescence, cells or 10 µm sections of frozen mouse mammary tissue were fixed with 4% paraformaldehyde, permeabilised in PBS +0.1% Triton-X and then blocked in FBT buffer (5% FBS, 1% BSA, 0.05% tween-20, 10 mM Tris pH 7.5, 100 mM MgCl_2_). Primary antibodies: mouse anti-human PTPRJ (1∶100), hamster anti-murine PTPRJ (1∶500) rabbit anti-beta-catenin (1∶100) (Sigma), mouse anti-E-cadherin (1∶100) (Transduction Laboratories), rabbit anti cytokeratin 5 (1∶500) (Covance) were diluted in FBT. Secondary antibodies were Alexa-488- or Alexa 594-conjugated secondary antibodies (Molecular Probes, OR, USA) were used at 1∶500 dilution. Nuclei were counterstained with Hoechst 33258 (Sigma) and F-actin was stained with a 1∶500 dilution of Rhodamine Phalloidin (Sigma). Samples were mounted in immunofluorescent mounting media (DAKO, CA, USA) and images were collected using a Zeiss LSM Confocal microscope at 630× magnification. Anti-murine Ptprj [43], was a gift from Prof. A. Weiss and Dr. Jing Zhu (Howard Hughes Medical Institute, Center for Arthritis, University of California San Francisco). PTPRJ expression in normal human and tumor samples were assessed by pathologists (ACV, LdS and SRL) and observations regarding localisation and frequency of positivity within each sample were made. The ER, PR and HER2 status of the tumors was assessed as part of routine hospital diagnosis of the corresponding paraffin blocks and this information was retrieved from pathology records. Tumors were considered positive if greater than 10% of cells stained with anti-ER (Novacastra, UK) and anti-PR (Novacastra, UK) antibodies. HER2 staining and scoring was according to the Herceptest (Dakocytomation, Denmark) protocol. Only cases with 3+ staining or with amplification using CISH were regarded as positive.

### Western Blotting

Cell lines were grown to sub-confluence, harvested and lysed in RIPA buffer (150 mM NaCl, 1% NP−40, 0.5% sodium deoxycholate, 0.1% sodium dodecyl sulfate, 50 mM Tris pH 7.4) containing 1 mM PMSF and protease inhibitor cocktail (Roche). After centrifugation, 30 µg of soluble protein was separated on SDS-PAGE, transferred to PVDF and probed with mouse anti-hPTPRJ 1∶100, hamster anti-mouse PTPRJ 1∶10000, rabbit anti-actin (Sigma-Aldrich, MO, USA) 1∶1000 or mouse anti-V5 tag (1∶2500) (Serotec, Oxford, UK) plus HRP-conjugated corresponding secondary antibody at 1∶2500 (Cell Signalling Technologies, Danvers, MA, USA) and visualised by ECL (Amersham, UK).

### Real-time PCR Analysis

RNA from homogenised cell pellets or ground mouse mammary gland or normal human breast tissue was extracted with Trizol or Qiagen RNeasy mini Kit [41]. cDNA for each sample was generated using Superscript III Reverse Transcriptase (Invitrogen, CA, U.S.A.) and diluted as appropriate. Primers included m*Ptprj* Forward 5′ CAG TAC AGT GAA TGG GAG CAC TGA C 3′; m*Ptprj* Reverse 5′ GTC CGT ATT CTG CCA CTC CAA CT 3′; *WAP* Forward 5′ GTG GTA GGA CCC GCA AAA CTC 3′; *WAP* Reverse 5′ CAC GGC CCG GTA CTA CTG AT 3′; m*Ptprj-as1* Forward 5′ GGA TCC TCA GAA CCC ATG AA 3′; m*Ptprj-as1* Reverse 5′ ACC GAC TGT CCA GTG AGA CC 3′; m*Ptprj-as1* Reverse 2 5′ TGA TTG AAG GAC AGC TGG AA 3′; h*PTPRJ* Forward 5′ CCT GAA GCC AGG GGT TCA ATA C 3′; h*PTPRJ* Reverse 5′ CCC GGC TTC TCT CTG TAT TGC 3′. Real-time PCR reactions were performed using SYBR Green PCR Master Mix in a 7900 Fast Real-time PCR System (Applied Biosystems, CA). Reactions were performed in triplicate for each sample and included no template and no reverse transcription negative controls. Relative expression was normalised to endogenous 18S rRNA [42] and a student’s t-test was performed to calculate statistical significance. For analysis of *PTPRJ* expression in the tumor and normal tissues of the Cancer survey panel (Origene), assays were performed in duplicate plates with pre-normalized (beta-actin) cDNA solubilized (15 min on ice) in a 10 µl reaction mix consisting of SYBR Green PCR Master Mix with 2.5 pmol of both h*PTPRJ* Forward 5′ CAC CAT CTC TCC AGA AGT GGA C 3′, and h*PTPRJ* Reverse 5′ GGC GTC ATC AAA GTT CTG CCA AC 3′ in a 7900 Fast Real-time PCR System (Applied Biosystems, CA) as above.

### Expression of *PTPRJ*

Full-length V5-tagged human *PTPRJ* cDNA was subcloned from pGene V5/His [11] into pBABE Hygro [44] and transfected into BOSC-32 packaging cells to produce ecotropic retroviruses. Filtered viral supernatant plus 4 µg/mL polybrene was used to infect recipient human breast cell lines expressing the cell surface receptor for mouse ecotropic retroviruses, and also the murine HC11 cell line. Cells were fixed and stained in crystal violet as an indicator of colony survival and protein lysates analysed for ectopic *PTPRJ* expression.

### HC11 Differentiation

Differentiation assays were performed as described previously [45]. Briefly, cells were grown to confluency over 3 days in the presence of EGF (10 ng/ml). EGF was removed and 24 hours later (Day 4) cells were treated with 10^–6^ M dexamethasone and 5 µg/ml ovine prolactin (oPRL) (Sigma) until Day 8. Domes were counted in triplicate wells at Day 8. Differentiation was further confirmed by beta-casein expression by RT-PCR [45].

## Results

### *PTPRJ* mRNA is Expressed in Normal Breast Tissue and Shows Downregulation in Breast Tumors

**Figure 1:**
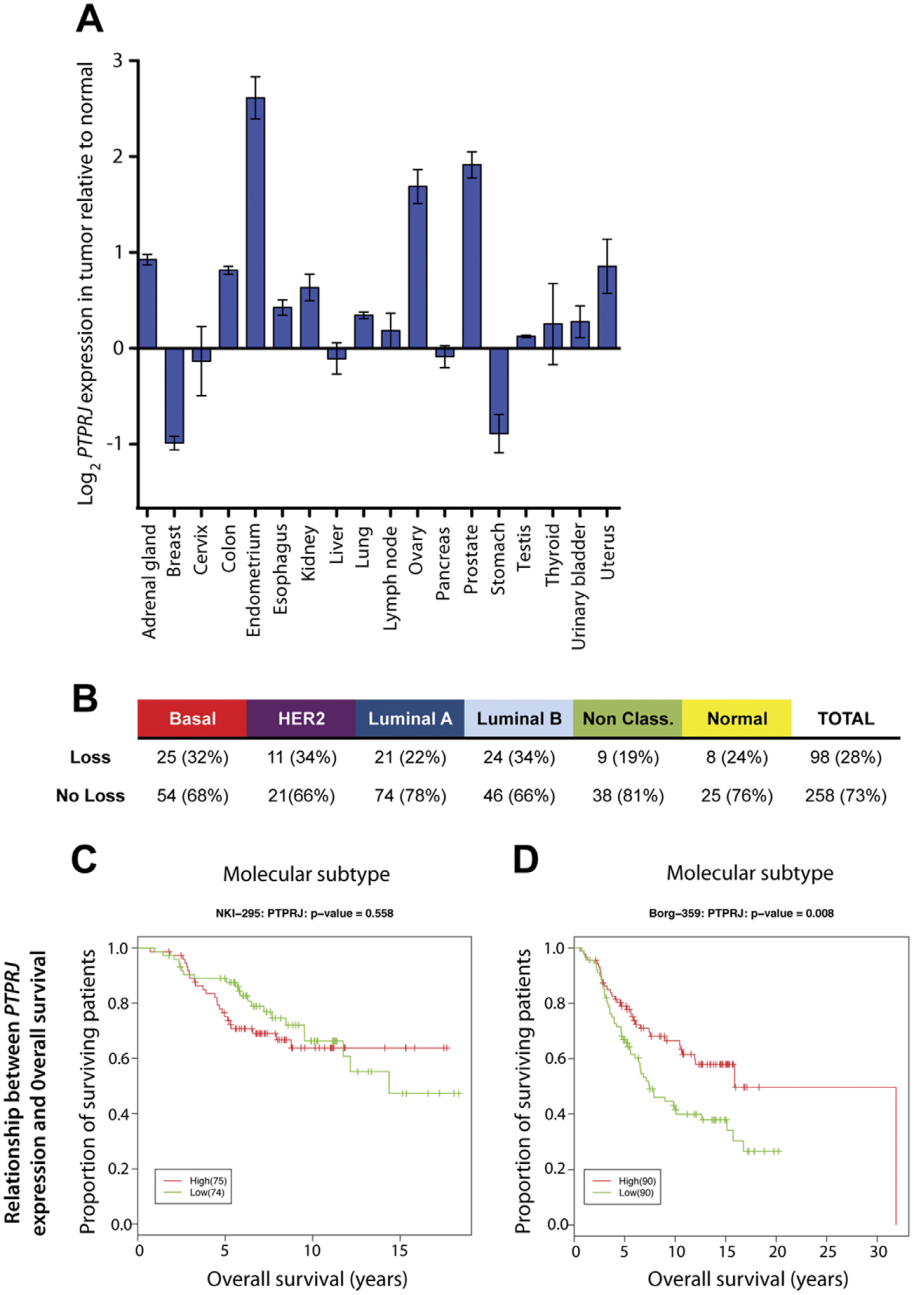
*PTPRJ* expression in tumors and meta-analysis of breast cancer cohorts. (A) Expression (log2) of *PTRPJ* in 18 cancer tissues relative to the corresponding normal tissue. Various numbers of biological replicates were included for each tissue from a combined total of 381 samples (OriGene). Expression was determined by quantitive PCR. (B) Number and proportion of genomic alterations at the *PTPRJ* locus (chromosome 11 at location 47,958,689–48,146,246) in Borg-359 breast cancer cohort [32]. Gain or loss was defined based on the segmentation mean that is either above 0.1 or below -0.1. Intermediate values were classed as copy neutral. Relationship between *PTPRJ* gene expression and overall survival time on both the © NKI-295 and (D) Borg-359 data sets. Kaplan-Meier survival curves between groups with High/Low expression in *PTPRJ*. Gene expression above the 75% quartile or below the 25% quartile of *PTPRJ* expression profiling was classified as “High” and “Low” respectively.

Analysis of baseline *PTPRJ* mRNA expression in a screening library of 18 normal human tissue types revealed pancreas, breast and liver tissue express the highest levels of *PTPRJ* whilst the lowest were found in endometrium, ovary and bladder (Fig. S1A). Interestingly, comparison of *PTPRJ* mRNA in tumors derived from each tissue type revealed the greatest relative downregulation in breast tumors (n = 23) (Fig. 1A), followed by stomach, consistent with the literature supporting a tumor suppressive role in these two tissues [2].

### *PTPRJ* Gene Copy Number and mRNA Expression in Breast Cancer

To address whether genomic alterations at the *PTPRJ* locus are common in the breast tumors above, the integrity of the *PTPRJ* locus was investigated by metaanalysis of a global DNA copy number study of 359 breast tumors [32]. In total, the gene region is lost in 98 (28%) of 356 informative breast cancers (Figure 1B) consistent with published loss of heterozygosity data from a much smaller cohort [2]. This genomic loss is not however associated with any particular molecular phenotype. Whilst the NKI-295 cohort showed no significant association between *PTPRJ* expression and overall survival (Figure 1C), patients with higher *PTPRJ* expression in the larger Borg-359 dataset with longer follow-up showed a greater better overall survival over the more than 20 year follow-up post-diagnosis (p = 0.008) (Figure 1D). Multivariate analysis adjusting for tumor grade however, showed lack of independence of *PTPRJ* expression as a prognostic factor in this data series (p = 0.293). Furthermore, no or only very weak associations were found between *PTPRJ* mRNA levels and tumor grade or molecular phenotype in two independent breast cancer cohorts (NKI-295 and Borg 359) (Figure S1B - E).

### PTPRJ is Localized at the Apical Surface of the Normal Human Breast Epithelia

**Figure 2:**
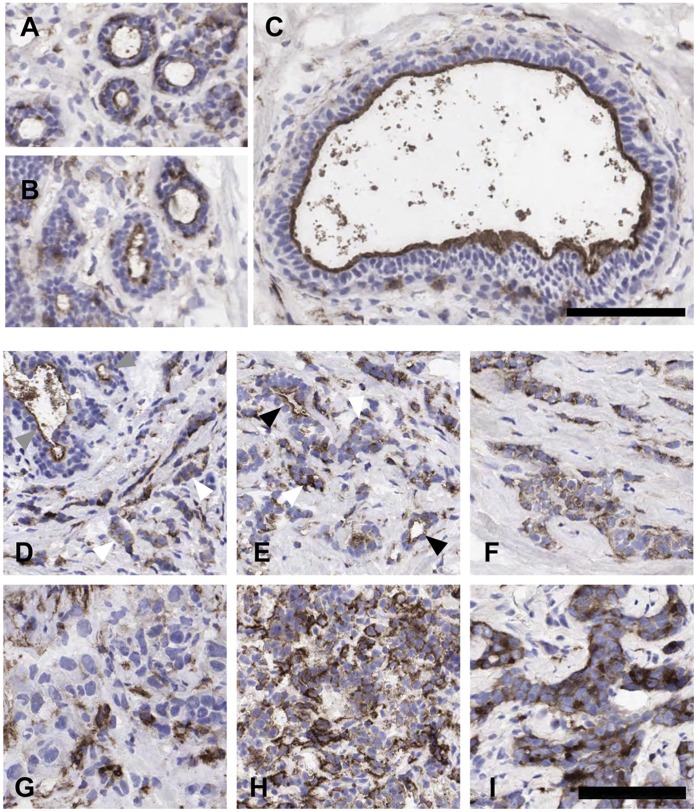
PTPRJ localization in normal and cancerous human breast. (A-C) Immunoperoxidase staining of PTPRJ in fresh frozen sections of normal human breast. Examples of alveoli (A,B) and a duct (C) are shown. Scale bar  = 100 µm. (D-I) PTPRJ localisation is heterogeneous in breast carcinoma, appearing dysregulated with tumor differentiation (loss of tubule formation). (D-F) Photomicrographs of PTPRJ IHC in different fields of the same specimen of grade 3 invasive ductal carcinoma. Grey arrows indicate normal adjacent tubules with apical staining of luminal cells. Black arrows indicate areas of the carcinoma where tubule architecture is retained and exhibit apical expression of PTPRJ in luminal cells. White arrows indicate nests of carcinoma cells where PTPRJ expression is diffuse and cytoplasmic, also shown in (F). (G-I) depict three poorly differentiated independent (grade 3) IDC that do not exhibit tubule formation. Scale bar  = 100 µm. Images were taken at 20× magnification.

*PTPRJ* is often reported as being ubiquitously expressed, yet its emerging role in macrophage membrane ruffling and in B and T cell activation indicates clear cellular and subcellular specific functions [20], [46], [47]. *In vitro*, *PTPRJ* regulates the integrity of intracellular junctions in epithelia [24]. To determine whether *PTPRJ* protein expression and localization is important for maintaining the epithelial integrity of the breast, immunohistochemistry (IHC) was performed on fresh frozen sections of normal human breast. PTPRJ predominantly localized to the apical surface of luminal epithelial cells of alveoli (Fig. 2A, B) and both small and large ducts (Fig. 2C). There were also sporadic immuno-positive cells in the basal layer and within the stroma (Fig. 2C). Where multiple layers of epithelia were present in ducts, only those cells adjacent to the lumina were positive, with the underlying suprabasal cells showing no evidence of *PTPRJ* expression (Fig. 2C). This compartment and subcellular specific pattern of apical expression suggests a role in luminal epithelial maintenance in the breast.

### PTPRJ Localization in Breast Carcinoma

To address whether *PTPRJ* protein expression and localisation is altered in breast cancer, IHC was performed on 7 µm sections of 27 frozen invasive ductal carcinoma (IDC) of mixed grade and ER/PR/HER2 status (described in Methods and Table S1). Although *PTPRJ* protein expression was detected in all cases (Fig. 2D-I), the proportion of positive cells and the distribution of staining were variable, as shown in panels 2D-E depicting different fields of view from the same grade 3 invasive ductal carcinoma. No clear relationship between the pattern of *PTPRJ* protein expression and other clinical data was observed in this cohort of tumors. However, in areas where the tumor retained partial glandular/tubular architecture, PTPRJ delineated the lumina (Fig. 2E, black arrowheads) i.e. showed the apical staining of luminal cells observed in normal breast samples (Fig. 2A-C) or in normal areas of tissue adjacent to the tumor (Fig. 2D, grey arrowheads). Where the tubular architecture was lost, diffuse cytoplasmic staining of PTPRJ was observed (Fig. 2E-I), and this was particularly prominent where there were nests of carcinoma cells (Fig. 2D, 2E, white arrows, and 2F). Most cases demonstrated cytoplasmic positivity in 30–50% of all tumor cells. Consistent with this observation, 15 of the 27 invasive carcinoma that completely lacked tubule formation exhibited only cytoplasmic PTPRJ staining (examples in Fig. 2G-I). Thus, although *PTPRJ* protein expression was not consistently lost in breast cancer, loss of apical staining of PTPRJ may be associated with the level of differentiation within the tumor.

### Endogenous *PTPRJ* Protein Expression and Localization Varies Amongst Human Breast Cancer Cell Lines

**Figure 3:**
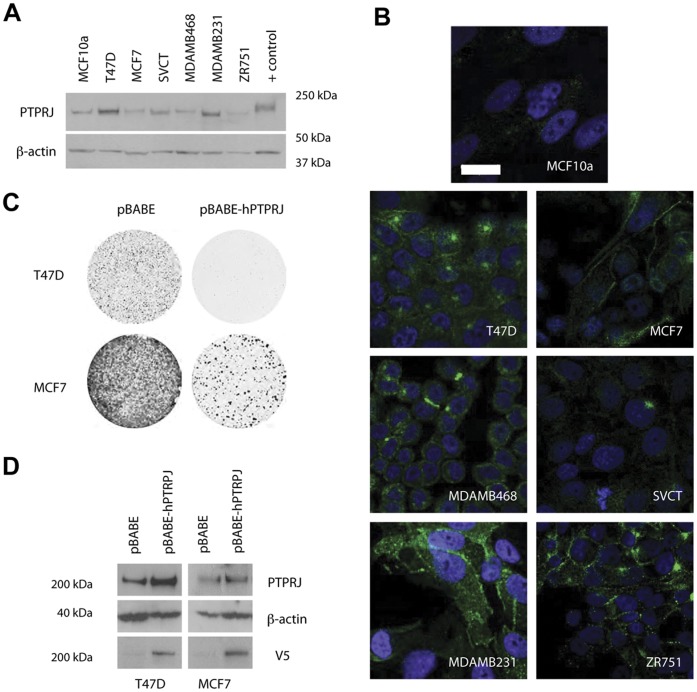
PTPRJ in human breast cancer cell lines. (A) Immunoblotting of PTPRJ in human breast cancer cell lines. Human macrophage cell line RAW 264.7 cells were used as a positive control (B) Localisation of endogenous PTPRJ in human breast cancer cell lines. Scale bar  = 20 µm. (C) Effect of PTPRJ expression on colony formation of human breast cancer cell lines. T47D and MCF7 cells were infected with (pBABE-hPTPRJ-V5) or without (pBABE) and colonies were fixed and stained with crystal violet. (D) Immunoblotting of transduced PTPRJ in human breast cancer cell lines.

The expression and localization of endogenous PTPRJ in a panel of human breast cancer cell lines was examined by western blotting and immunofluorescence. Consistent with the tumor data, *PTPRJ* protein was detected in all cell lines, with expression levels varying from highest in T47D and MDAMB231 cells, to lower in MCF7, ZR751 and MCF10A cells (Fig. 3A). This pattern largely reflected *PTPRJ* transcript levels detected by qPCR (Fig. S2). Interestingly, in addition to a range of expression levels, *PTPRJ* protein localization patterns were also highly variable amongst the different breast cancer cell lines, with perinuclear staining in T47D cells and SVCT cells; intermittent staining of cell-cell contacts in MDAMB468 cells; smooth or punctate plasma membrane staining in MCF7 and ZR751 cells respectively; and punctate cytoplasmic staining in MDAMB231 and SVCT cells (Fig. 3B). Costaining for F-actin which assists in showing the cell boundaries, revealed that even where neighbouring cells are in close contact, PTPRJ localisation is not necessarily membranous (Fig. S3). As plasma membrane localisation of PTPRJ is critical for its biological function [23] the variability of PTPRJ localization might confer variable endogenous PTPRJ function in these *in vitro* models.

Since the overall levels of *PTPRJ* varied in the breast cancer lines, we tested whether ectopic expression of wildtype *PTPRJ* affected phenotype. T47D, MCF7 and MDAMB231 (data not shown) lines were chosen as representative of cells with high, low and intermediate endogenous levels of *PTPRJ* respectively. Retroviral transduction of V5-tagged human *PTPRJ* (Fig. 3C) had a similar effect in all these cells, with ectopic expression of *PTPRJ* significantly reducing colony formation independent of the endogenous levels (Fig. 3D).

### Ptprj is Differentially Regulated during Mammary Development and Exhibits Apical Localization in Luminal Mammary Epithelium of Pregnant Mice

**Figure 4:**
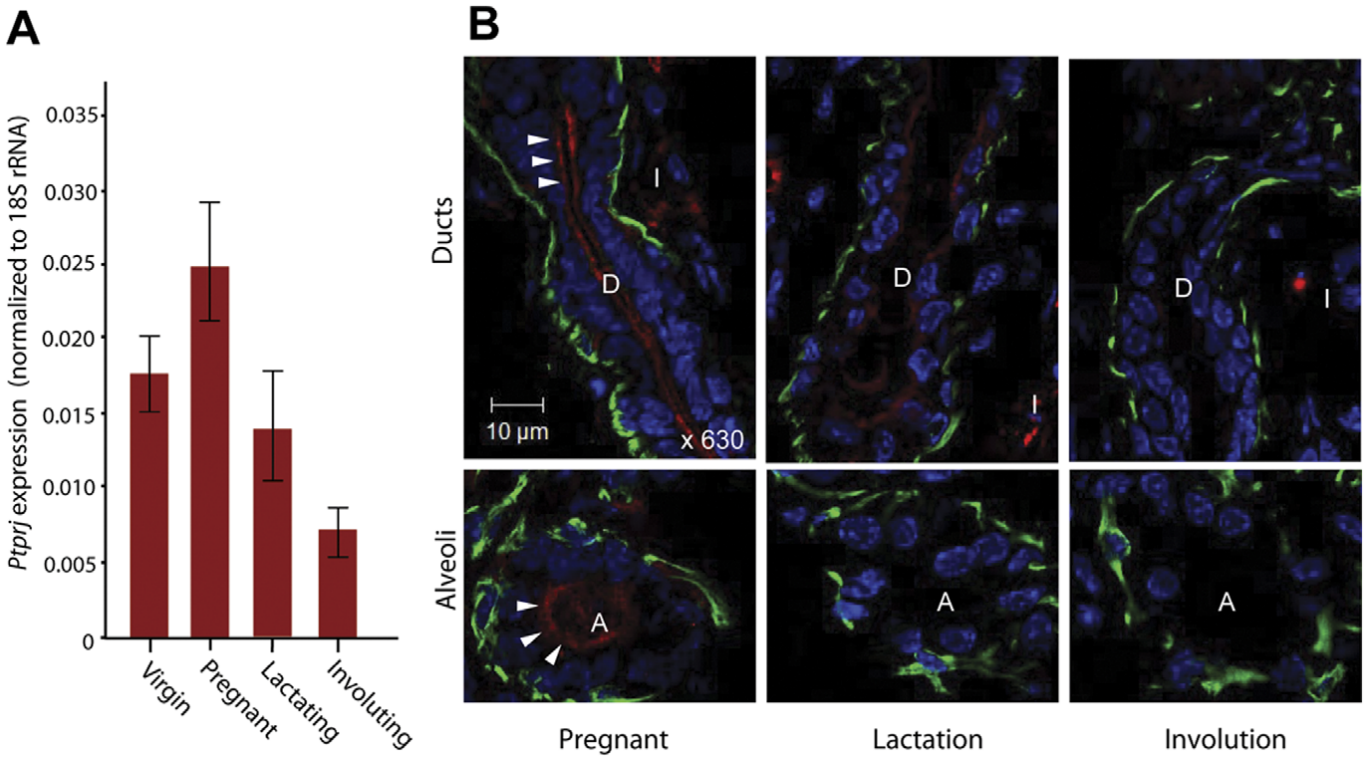
Expression and localization of endogenous *Ptprj* and *Ptprj-as1* in the mouse mammary gland. (A) qPCR of *Ptprj* and *Ptprj-as1* in cDNA from mammary glands extracted from nulliparous (virgin) mice, day 14 of pregnancy, day 1 of lactation and day 2 of involution relative to 18S rRNA. Error bars represent SEM, n = 4. (B) Immunofluorescence of Ptprj in frozen sections of mouse mammary gland during pregnancy, lactation and involution. Ptprj was detected with hamster-anti- Ptprj/Alexa-546-anti-hamster IgG (red), costained with rabbit anti-keratin 5/Alexa 488-anti-rabbit IgG Alexa-488 (green) and Hoescht nuclear counterstain (blue). White arrows show where Ptprj delineates the lumen of ducts (D) and alveoli (A). (I)  =  interstitial tissue. Scale bar  = 10 µm.

To determine whether *Ptprj* is differentially regulated during lactational development, *Ptprj* expression levels were determined by quantitive PCR on total RNA isolated from virgin, pregnant, lactating and involuting murine mammary gland. *Ptprj* levels were found to be significantly higher during pregnancy compared to adult virgin and lactating glands and were lowest during involution (Fig. 4A).

To investigate whether the apical localisation of PTPRJ was conserved in the mouse mammary gland and to further characterize *Ptprj* expression *in vivo*, immunofluorescent staining of Ptprj was performed on frozen sections of mammary gland from pregnant, lactating and involuting mice. Ptprj was found to be most prominent on the apical surfaces of luminal cells lining the ducts of mammary glands from pregnant mice (Fig. 4B, upper left panel, arrowheads), reminiscent of our results with human PTPRJ (Fig. 2). Alveoli in pregnant mouse mammary gland also displayed apical staining (Fig. 4B, lower left panel, arrowheads), a feature lost in the alveoli in lactating and involuting mouse mammary glands (Fig. 4B middle and right panels). Sporadic stromal staining was observed, consistent with staining of Ptprj-positive macrophages [48]. Conservation of apical localisation and dynamic regulation throughout mammary lactational development further support the hypothesis that appropriate protein localisation is important for maintaining the epithelial integrity of the breast.

### Identification of Intronic and Antisense Long Noncoding RNAs, within the Mouse *Ptprj* Locus

Increasing numbers of noncoding RNAs have been identified that are functionally related to nearby protein-coding genes [49], [50], [51], [52]. Therefore, to identify long ncRNAs that may be involved in *Ptprj* regulation during mammary development, we examined microarray expression profiling data of mammary epithelial cells derived from pregnant, lactating and involuting mice [51]. Within this dataset, we identified seven probes that targeted long ncRNAs that originated from the *Ptprj* locus (Table S2 and Fig. S4). All of these long ncRNAs occurred within the first intron of *Ptprj*; two were on the antisense strand and the remaining five were on the sense strand (Fig. S4). None of the 7 probes were signficantly differentially expressed between the different developmental stages of the mammary epithelial cells. Although it remains a possibility that these lncRNAs are involved in PTPRJ regulation, it is unlikely that they are involved in mammary gland development and therefore were not further investigated.

### *Ptprj* Regulates Murine Mammary Epithelial Differentiation *in vitro*

**Figure 5:**
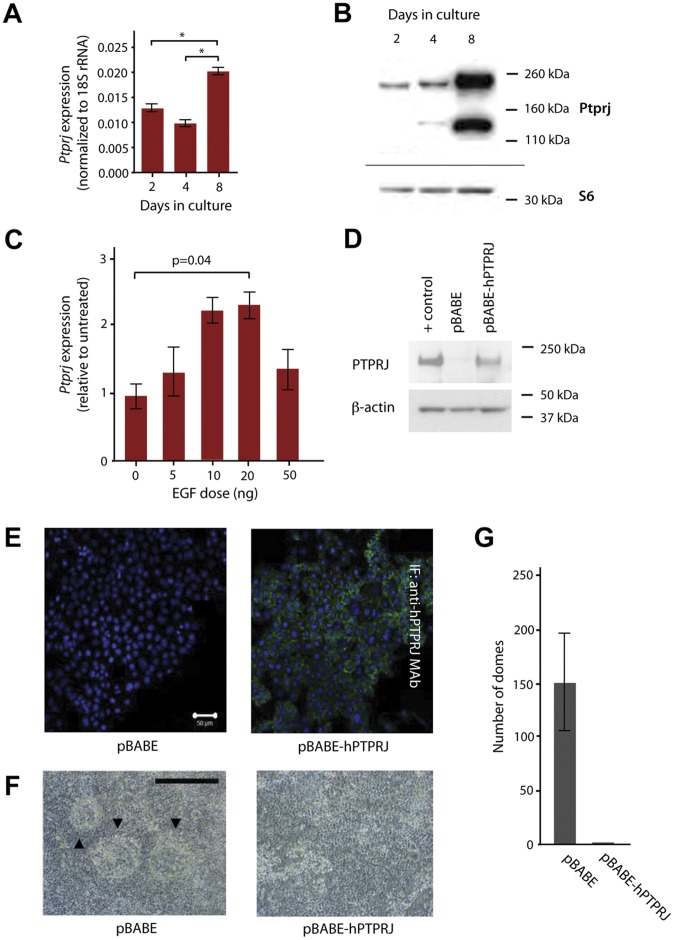
*Ptprj in vitro* differentiation assays. (A) qPCR of endogenous *Ptprj* expression in HC11 cells during *in vitro* differentiation. Bars represent SEM of 3 technical replicates. * p<0.001. (B) Immunoblotting of *Ptprj* protein levels during HC11 differentiation. (C) *Ptprj* and *Ptprj-as1* expression in HC11 EGF dose response assay. RNA expression was quantified by qRT-PCR (normalized to 18S rRNA) and expressed as fold change compared with untreated control at day 2. Error bars represent SD, n = 3. There was a statistically significant increase of *Ptprj* (p = 0.02) between 0 ng and 20 ng and a significant decrease of *Ptprj* (p = 0.02) between 20 ng and 50 ng. (D) Immunoblotting of retrovirally transduced human *PTPRJ* in HC11 cells. MDA-MB-231 cells were used as a positive control. **(**E) Immunofluorescence of HC11 cells in the absence (pBABE) and presence of retrovirally-transduced human *PTPRJ* (pBABE-hPTPRJ-V5). Scale bar  = 50 µm. (F) Morphology of dome formation during *in vitro* differentiation of HC11 cells in the presence and absence of retrovirally-transduced hPTPRJ-V5. Scale bar  = 500 µm. (G) Effect of retrovirally-transduced hPTPRJ-V5 on dome formation in a representative experiment. Error bars represent SEM.

*In vitro*, murine mammary HC11 cells can differentiate under the appropriate lactogenic stimulus [45], [53], to form 3-dimensional dome structures. To investigate the expression of *Ptprj* during *in vitro* differentiation of mammary epithelial cells, quantitative RT-PCR and western blotting were performed during the proliferative (days 2 and 4) and differentiation (day 8) phases of this *in vitro* assay. *Ptprj* mRNA and protein levels both increased significantly by day 8 compared to days 2 and 4 (Fig. 5A, B).

Failure to withdraw EGF in this cell line inhibits full differentiation [54]. We also know from investigations of the relationship between PTPRJ and EGFR, that one proposed tumour suppressive mechanism for DEP-1 is the inactivation and retention of EGFR at the cell membrane and therefore the abrogation of EGF-induced signaling [16]. We hypothesised that the increase in *Ptprj* expression observed in the 8 day HC11 assay may be a result not only of the introduction of lactogenic hormones, but also the EGF withdrawal, which occurs at day 2. To examine the effect of EGF on *Ptprj* expression, we performed a dose response assay and revealed a significant increase in *Ptprj* expression (p = 0.02) providing evidence of EGF signalling controlled regulation of *Ptprj*.

The developmental regulation of *Ptprj in vivo* and *in vitro* raised the possibility that *Ptprj* could play a role in the differentiation of mammary gland cells. To examine this, HC11 cells were transduced with wildtype human *PTPRJ*-containing retrovirus (Fig. 5D, E) that clearly localizes to the plasma membrane. *PTPRJ*-expressing cells showed a dramatic reduction in dome formation in response to lactogenic hormones, producing at most one dome per well compared with up to 200 domes per well in cells infected with control retroviral particles (Fig. 5F, G) with no associated change in HC11 cell proliferation rate or cell morphology (Figure S5 and Fig. 5E). This dramatic effect suggests that *in vitro* dome morphogenesis is very sensitive to an altered balance of *PTPRJ* expression.

## Discussion

*PTPRJ* is recognized as a tumor suppressor gene in colorectal carcinoma [2] but to date neither its role in normal development or tumorigenesis had been thoroughly investigated in breast. Overall, our *in silico*, *in situ* and *in vitro* findings indirectly support a tumor-suppressive role for this gene. Meta-analyses of a large breast cancer cohort revealed frequent loss of the *PTPRJ* locus, consistent with a tumor suppressor function and confirming loss of heterozygosity findings in a smaller cohort [2]. Consistent with this, *PTPRJ* transcript expression analysis in a tumor library suggests reduced expression in breast tumors compared to normal. Furthermore, the association between low *PTPRJ* levels on poorer survival in the Borg-359 cohort are also suggestive of tumor suppressive behaviour. Although the relationship between PTPRJ expression and survival was not significant in the NKI-295 study, this could be accounted for by the smaller number and shorter duration of this study follow-up, the different clinicopathological characteristics of patients analysed, including different median age (NKI-295 subjects were all under 53 years old whereas the Borg 359 cohort covered a more variable age range of 22-88 yrs) and the distribution of histological grading, estrogen receptor status and lymph node status.

Different molecular subtypes of breast cancer exhibit different overall frequencies of copy number alterations, implying these subtypes develop along distinct genetic pathways [55], [56]. Many studies now focus on unraveling subtype specific pathogenesis, Whilst we found genomic loss of *PTPRJ* to be frequent, we observed no subtype specific pathogenesis or transcriptional expression. Therefore, although our findings are consistent with a tumor suppressor role for PTPRJ, *PTPRJ* loss does not appear to be driving a particular breast cancer phenotype.

PTPRJ protein was mislocated in the majority of primary breast tumors, which would most likely result in impaired function of the phosphatase, as concluded by other studies investigating its localisation [23]. To our knowledge this is the first report of normal PTPRJ localisation patterns in the human (and mouse) mammary gland, revealing a strong apical staining of the luminal epithelial layer of the tissue. Within the same breast tumor specimen, normal apical PTPRJ staining was retained where tubule formation was conserved and was diffuse or cytoplasmic in areas where this architecture was lost. Whilst the sample numbers in the study are small, a greater proportion of the grade 3 breast carcinoma exhibited exclusive cytoplasmic staining compared to grade 2, consistent with the loss of PTPRJ apical localisation concurrent with lack of tubular architecture. This is reminiscent of MUC1 which is dysregulated during tumorigenesis. [57], and is currently being pursued as a candidate for targeted therapies [58], [59], Therefore, mislocalisation of PTPRJ may mediate altered cellular behaviour in a similar manner.

As the patterns of PTPRJ protein localisation varied between normal and cancerous breast, it was not possible to conclude whether overall protein levels were also reduced in tumors or whether the mislocalisation of PTPRJ is a cause or a consequence of tumorigenesis. Although it has previously been assumed that PTPRJ localisation in epithelial cell lines is primarily at the plasma membrane, our analysis of both luminal- and basal-like breast epithelial cell lines unexpectedly also revealed variable expression and localisation of the PTPRJ protein, compatible with our human breast cancer tumor analyses. Perinuclear staining of PTPRJ was observed in T47D, ZR751 and SVCT cell lines and punctate intracellular staining was seen in MDAMB231 cells. In MCF7 cells, PTPRJ localisation was at the primarily plasma membrane, similar to that previously observed [11], but MDAMB468 showed a very peculiar pattern of intermittent intense staining of cell-cell contacts. These observations indicate that in addition to transcriptional and post-transcriptional control [60] there is a further level of complexity in *PTPRJ* regulation: appropriate intracellular localisation [23]. Since PTPRJ directly interacts with tight junction proteins and plays a role in epithelial barrier permeability [24], its function and the effect of identified SNPs of PTPRJ in regions that may affect localisation, cell polarity and transformation warrant further investigation [2].

The apical pattern of expression of *PTPRJ* was also conserved in the mouse mammary gland, particularly during pregnancy where increased antibody staining correlated with an increase in mRNA. The changes in *PTPRJ* mRNA and protein during different developmental stages of the mammary gland *in vivo* and *in vitro* indicate that its expression and localisation are sensitive to the differentiation status of the tissue It is possible that Ptprj expression is hormonally regulated, by, for example progesterone, whose levels drop during differentiation [61]. Stromal cells with the morphology of tissue macrophages also stained positively for PTPRJ, consistent with previous reports [48].

*In vitro,* PTPRJ expression reduced HC11 dome formation without affecting proliferation or survival, indicating that it may play an active role in this process. Since development of the apical surface is considered the final phase of acinar morphogenesis [62], forced alterations to the expression of an apical protein such as PTPRJ that interacts with occludin and ZO-1 may affect the 3-dimensional formation of the acinus [24]. Since PTPRJ increases during differentiation, one might expect overexpression of PTPRJ to increase rather than diminish dome formation. It is possible that the increase in Ptprj expression observed in the 8 day HC11 assay is a result not only of the introduction of lactogenic hormones, but also the EGF withdrawal, which occurs at day 2. Failure to withdraw EGF in this cell line inhibits full differentiation [54]. We also know from investigations of the relationship between PTPRJ and EGFR, that one proposed tumour suppressive mechanism for DEP-1 is the inactivation and retention of EGFR at the cell membrane and therefore the abrogation of EGF-induced signaling [16]. To examine the effect of EGF on PTPRJ expression, we performed a dose response assay and revealed a significant increase in PTPRJ expression (p = 0.02), nonetheless providing evidence of EGF signalling controlled regulation of PTPRJ. Inappropriate ectopic overexpression of hPTPRJ may also compete in some way with endogenous mPtptrj, but interact with a different subpopulation of substrates. Whilst overexpression studies may lead to artefacts, for example due to aberrant signaling, our results indicating that ectopic expression of PTPRJ leads to a reduction in colony formation in breast cancer cell lines (Fig 3C) is consistent with our previous results and those of Trapasso et al and Keane et al [6], [8], [11]. Repeated attempts using different SiRNA oligonucleotides to knock down PTPRJ levels were unsuccessful, possibly due to the low transfection efficiency of the cells. Nevertheless, our data provides a good foundation for future studies examining the mechanism by which PTPRJ plays a role in these processes.

Although a *PTPRJ*/GFP knockout/knock-in mouse is embryonic lethal, two *PTPRJ* knockout mice lines show little observable phenotype [47], [63], [64]. In B cells and monocytes PTPRJ positively regulates cell function by activating *src* family tyrosine kinases [47] In contrast, PTPRJ has negative regulatory role in fibroblasts and other cell types [11] inhibiting growth factor stimulated signalling and proliferation. Thus the cellular background, localisation and access to specific substrates may determine whether PTPRJ enhances or inhibits cellular responses in different tissues. From this current study, the identification of long ncRNA that are expressed from within the first intron and the antisense strand of the Ptrprj gene may help further elucidate aspects of *Ptprj* regulation. lncRNAs regulate diverse mechanisms ranging from alternative splicing to epigenetic modification [65]. Further functional studies will be necessary to determine whether the function of lncRNAs identified in the Ptprj locus.

In summary, we have shown that *PTPRJ* is frequently lost in breast cancer, its expression is lower than normal breast and that low expression correlates with poorer overall survival. PTPRJ has a distinct apical localisation in mammary tissue and is regulated during murine mammary gland differentiation, *in vitro* and *in vivo.* Apical localisation pattern was lost with loss of tubule architecture in invasive ductal carcinoma although all tumors retained some protein expression. In oncogenic breast cell lines, varying expression levels and patterns of intracellular distribution were identified. Furthermore, over-expression in a mammary epithelial cell line inhibited *in vitro* differentiation, which suggests a role for PTPRJ in normal mammary gland development. Finally, our findings suggest altered subcellular localisation of PTPRJ as an additional regulatory factor in normal breast biology and oncogenesis.

## Supporting Information

Figure S1 ***PTPRJ*** **expression in normal and tumor tissues** (**A**) Expression (log2) of *PTPRJ* in 18 normal human tissues relative to a pooled average. Relationship between *PTPRJ* gene expression and histological grade (B-C) and molecular subtype (D-E) in both the NKI-295 [31] and Borg-359 data sets. (B-E) Either ANOVA or Kruskal-Wallis test was used to detect the difference in gene expression expression across different histological grades or molecular subtypes. Molecular subtypes were identified using Hu’s SSP [33] (D) or Borg [32] (E).
(TIF)

Figure S2 **Real time PCR analysis of** ***PTPRJ*** **expression in breast epithelial cell lines and normal human breast samples.** Graph depicts mean expression of *PTPRJ* relative to 18S rRNA. Error bars represent SEM of three technical replicates.
(TIF)

Figure S3 **Immunofluorescent detection of PTPRJ and F-actin in breast cancer cells lines.** Costaining using anti-PTPRJ antibody, Phalloidin stain and Hoescht nuclear counterstain was performed on subconfluent cultures of MCF7, MDAMB468, SVCT and MDAMB231 cells. Frames showing areas with frequent cell-cell contact are shown.
(TIF)

Figure S4 **Genomic context of** ***Ptprj*** **and associated long noncoding RNAs (lncRNAs) in mouse.** Arrows indicate the direction of transcription. Binding sites of microarray probes referred to in Table S are shown in dark blue.
(TIF)

Figure S5 **Effect of ectopic hPTPRJ on HC11 cell proliferation.** HC11 cells transfected with pBABE and pBABE hPTPRJ were seeded into 96 well plates. After 24 hours when the cells had settled in the dish the baseline time-point to control for starting cell number was fixed in 4% PFA. Cells were then incubated in growth media and then fixed after 1 and 2 days. Fixed cells were stained with crystal violet for 5 minutes and then washed thoroughly in water and dried. Stain was eluted in 10% acetic acid and transferred to a clean multiwell plate.Absorbance at 595 nm was read and the data was normalised to the starting cell number by calculating absorbance relative to the 0 day time point. The slope between the absorbance readings at 48 and 24 hours was calculated. These values were then calculated relative to the pBABE vector only control in each assay to give a growth index. The assay was repeated three times and the average of the 3 relative values are given with standard deviation. A student’s t-test demonstrated that there was no significant difference between the proliferation rates of pBABE and pBABE h-PTPRJ cells between 24 and 48 hours.
(TIF)

Table S1 **Histological grading and characterization of frozen breast tumor samples.** Frozen sections were stained for eostrogen receptor (ER), progesterone receptor (PR), human epidermal growth factor receptor 2 (HER2) and PTPRJ (DEP-1) as described in materials and methods section.
(PDF)

Table S2 **Long noncoding RNA associated with the mouse PTPRJ locus.** Expression in mammary epithelium and relative nuclear to cytoplasmic enrichment was determined as described in Askarian-Amiri et al [51].
(TIFF)
