# Supplementary figures and images for: Expression and Function of the Protein Tyrosine Phosphatase Receptor J (PTPRJ) in Normal Mammary Epithelial Cells and Breast Tumors
Source: PLoS One. 2012 Jul 17;7(7):e40742. doi: 10.1371/journal.pone.0040742 (PMC3398958; doi:10.1371/journal.pone.0040742)

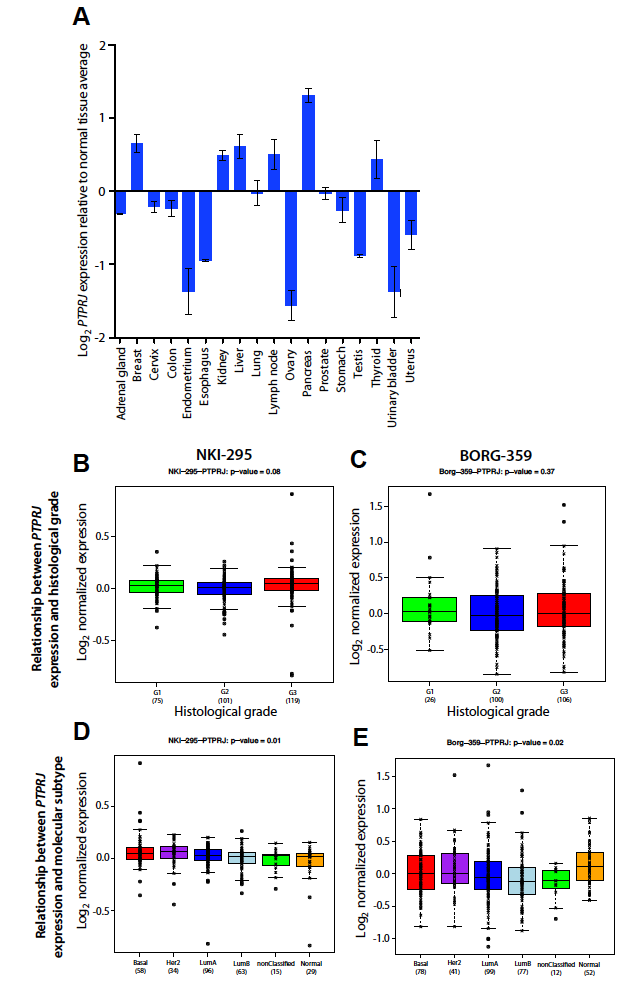

Supplement: Figure S1 — PTPRJ expression in normal and tumor tissues (A) Expression (log2) of PTPRJ in 18 normal human tissues relative to a pooled average. Relationship between PTPRJ gene expression and histological grade (B-C) and molecular subtype (D-E) in both the NKI-295 [31] and Borg-359 data sets. (B-E) Either ANOVA or Kruskal-Wallis test was used to detect the difference in gene expression expression across different histological grades or molecular subtypes. Molecular subtypes were identified using Hu’s SSP [33] (D) or Borg [32] (E). (TIF) [file pone.0040742.s001.tif]

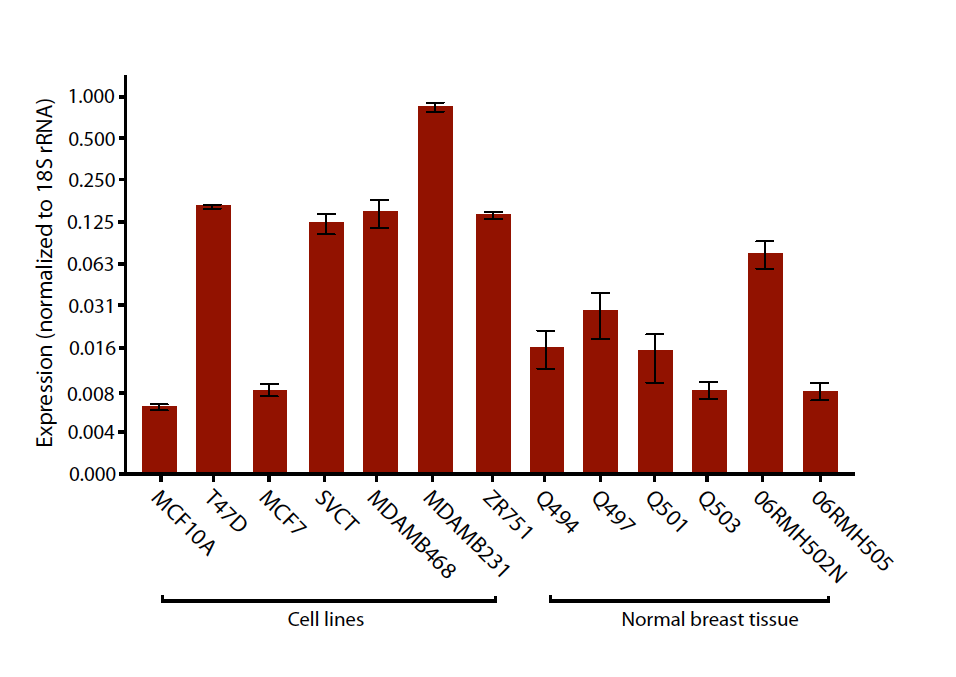

Supplement: Figure S2 — Real time PCR analysis of PTPRJ expression in breast epithelial cell lines and normal human breast samples. Graph depicts mean expression of PTPRJ relative to 18S rRNA. Error bars represent SEM of three technical replicates. (TIF) [file pone.0040742.s002.tif]

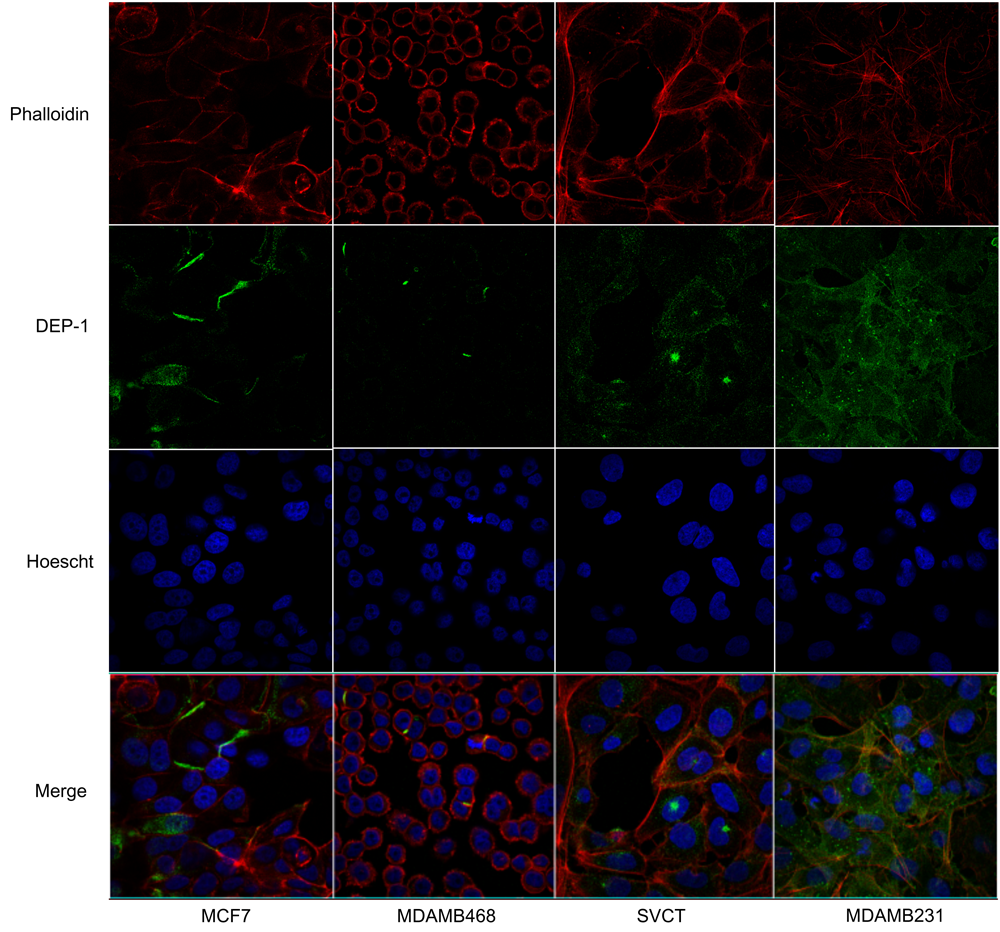

Supplement: Figure S3 — Immunofluorescent detection of PTPRJ and F-actin in breast cancer cells lines. Costaining using anti-PTPRJ antibody, Phalloidin stain and Hoescht nuclear counterstain was performed on subconfluent cultures of MCF7, MDAMB468, SVCT and MDAMB231 cells. Frames showing areas with frequent cell-cell contact are shown. (TIF) [file pone.0040742.s003.tif]

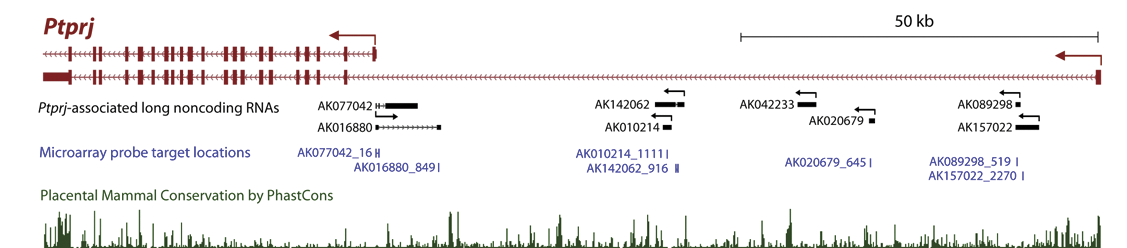

Supplement: Figure S4 — Genomic context of Ptprj and associated long noncoding RNAs (lncRNAs) in mouse. Arrows indicate the direction of transcription. Binding sites of microarray probes referred to in Table S are shown in dark blue. (TIF) [file pone.0040742.s004.tif]

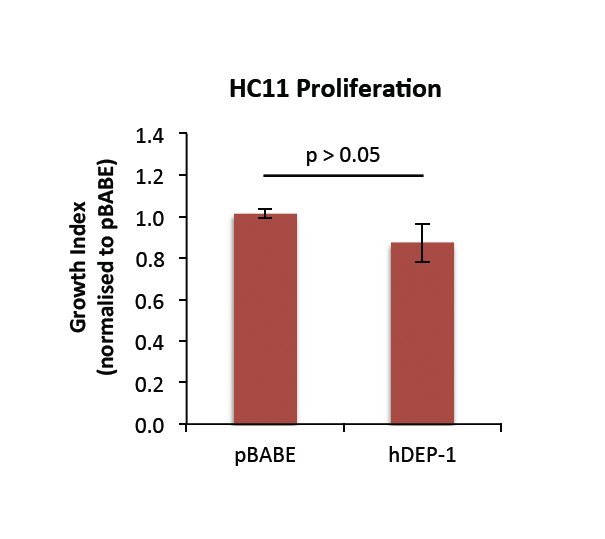

Supplement: Figure S5 — Effect of ectopic hPTPRJ on HC11 cell proliferation. HC11 cells transfected with pBABE and pBABE hPTPRJ were seeded into 96 well plates. After 24 hours when the cells had settled in the dish the baseline time-point to control for starting cell number was fixed in 4% PFA. Cells were then incubated in growth media and then fixed after 1 and 2 days. Fixed cells were stained with crystal violet for 5 minutes and then washed thoroughly in water and dried. Stain was eluted in 10% acetic acid and transferred to a clean multiwell plate.Absorbance at 595 nm was read and the data was normalised to the starting cell number by calculating absorbance relative to the 0 day time point. The slope between the absorbance readings at 48 and 24 hours was calculated. These values were then calculated relative to the pBABE vector only control in each assay to give a growth index. The assay was repeated three times and the average of the 3 relative values are given with standard deviation. A student’s t-test demonstrated that there was no significant difference between the proliferation rates of pBABE and pBABE h-PTPRJ cells between 24 and 48 hours. (TIF) [file pone.0040742.s005.tif]

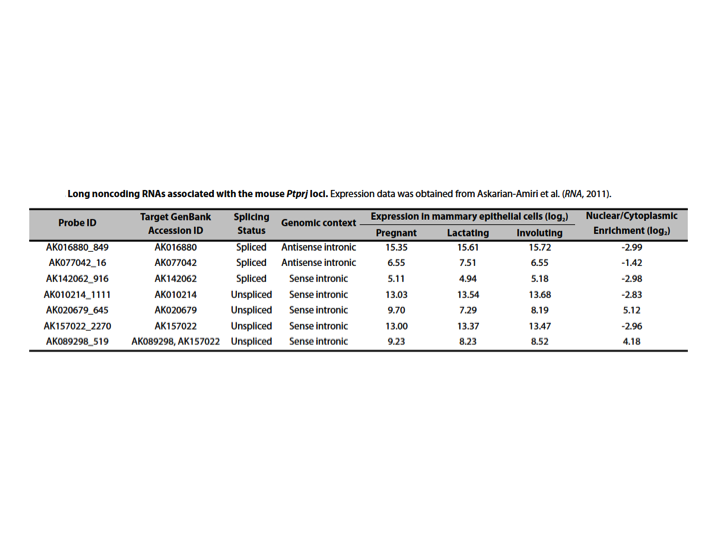

Supplement: Table S2 — Long noncoding RNA associated with the mouse PTPRJ locus. Expression in mammary epithelium and relative nuclear to cytoplasmic enrichment was determined as described in Askarian-Amiri et al [51]. (TIFF) [file pone.0040742.s007.tif]
