# Supplementary material for: Expression and Function of the Protein Tyrosine Phosphatase Receptor J (PTPRJ) in Normal Mammary Epithelial Cells and Breast Tumors
Source: PLoS One. 2012 Jul 17;7(7):e40742. doi: 10.1371/journal.pone.0040742 (PMC3398958; doi:10.1371/journal.pone.0040742)
Supplement: Table S1 — Histological grading and characterization of frozen breast tumor samples. Frozen sections were stained for eostrogen receptor (ER), progesterone receptor (PR), human epidermal growth factor receptor 2 (HER2) and PTPRJ (DEP-1) as described in materials and methods section. (PDF) [file pone.0040742.s006.pdf]

Supplementary Data Table 1

Supplementary data

Table of frozen breast tumour specimens examined for DEP-1 expression

Sample	Diagnosis	Grade	ER	PR	HER2	IHC+	Pattern
25	IDC	1	+	+	-	30-50%	⊙
78	IDC (Apocrine)	1	-	-	-	30-50%	●
152	IDC	2	+	+	-	30%	⊙
272	IDC	2	+	+	-	20-30%	⊙
73	IDC	2	+	+	-	80%	O
154	IDC	2	+	-	-	80%	⊙
137	IDC	2	+	-	-	30-50%	⊙
248	Mixed IDC ILC	2	+	+	-	30-40%	●
132	IDC	3	+	+	-	33%	●
200	IDC	3	+	+	-	30-50%	⊙
31	IDC	3	+	+	-	70%	⊙
46	IDC	3	+	+	-	30-50%	⊙
47	IDC	3	+	+	-	60%	●
48	IDC	3	+	+	-	20%	●
83	IDC	3	+	+	-	60-70%	●
145	IDC	3	+	+	-	80%	●
211	IDC	3	+	+	-	30%	●
244	IDC	3	+	+	-	30-80%	⊙
267	Mixed IDC ILC	3	+	-	-	80%	●
27	IDC	3	+	+	+	60%	●
32	IDC	3	+	+	+	30%	●
89	IDC	3	-	-	+	20-30%	●
91	IDC (Metaplastic)	3	-	-	+	40%	⊙
179	IDC	3	+	+	+	30-50%	⊙
118	IDC	3	-	-	-	90%	●
70	IDC	3	-	-	-	30%	●
299	IDC	3	-	-	-	50%	●

Histopathological diagnoses are given with histological grade, ER, PR and HER2 status as determined from hospital diagnostic pathological data on FFPE tissues as described in materials and methods.

IHC+ refers to the range of percentages of cells which stained immunopositive for DEP-1 on frozen section

Pattern refers to the distribution of DEP-1:

O apical expression only

● cytoplasmic staining only

⊙ cytoplasmic staining found together with apical staining in well differentiated areas within the same specimen
